# Physiological changes and symptoms associated with short-term exposure to electromagnetic fields: a randomized crossover provocation study

**DOI:** 10.1186/s12940-022-00843-1

**Published:** 2022-03-08

**Authors:** Po-Chang Huang, Jui-chin Chiang, Ya-Yun Cheng, Tain-Junn Cheng, Chien-Yuan Huang, Ya-Ting Chuang, Ti Hsu, How-Ran Guo

**Affiliations:** 1 Department of Environmental and Occupational Health, College of Medicine, National Cheng Kung University, 138 Sheng-Li Road, Tainan, 70428 Taiwan; 2 Department of Family Medicine, Chang Gung Memorial Hospital-Kaohsiung Medical Center, Chang Gung University College of Medicine, Kaohsiung, Taiwan; 3 Department of Occupational Medicine, Chi-Mei Medical Center, Tainan, Taiwan; 4 Tainan Science-Based Industrial Park Clinic, Chi-Mei Medical Center, Tainan, Taiwan; 5 Department of Occupational and Environmental Medicine, National Cheng Kung University Hospital, Tainan, Taiwan; 6 Occupational Safety, Health, and Medicine Research Center, National Cheng Kung University, Tainan, Taiwan

**Keywords:** Blood pressure, Cell phone, Heart rate, Heart rate variability, Hypersensitivity, Idiopathic environmental intolerance

## Abstract

**Background:**

The biological association between electromagnetic fields (EMF) and idiopathic environmental intolerance attributed to EMF (IEI-EMF) has not been established. To assess the physiological changes and symptoms associated with exposure to EMF, we conducted a randomized crossover provocation study.

**Methods:**

We recruited 58 individuals with IEI-EMF (IEI-EMF group) and 92 individuals without IEI-EMF (control group). In a controlled environment, all participants received EMF signals mimicking those from mobile phone base stations in a randomized sequence under the blinded condition. During the course, participants reported their symptoms and whether they perceived EMF, and we monitored their physiological parameters, including blood pressure (BP), heart rate (HR), and HR variability.

**Results:**

The IEI-EMF and control groups reported similar frequencies of symptoms during both the provocation and sham sessions. No participant could accurately identify the provocation. In both groups, physiological parameters were similar between the two sessions. The control group, but not the IEI-EMF group, had elevated HR when they perceived EMF exposure.

**Conclusions:**

No symptoms or changes in physiological parameters were found to be associated with short-term exposure to EMF, and no participant could accurately detect the presence of EMF. Moreover, the participants in the control group, but not those in the IEI-EMF group, had elevated HR when they perceived EMF.

## Background

The widespread use of electronic appliances, such as microwaves, televisions, and mobile phones, has raised the concern of the possible health effects of chronic exposure to the electromagnetic fields (EMF) emitted by these appliances. Symptoms such as headaches, fatigue, heightened stress levels, sleep disorders, skin-related sensations (e.g., burning, prickling, and itching), rashes, muscle pains and aches, and other health problems [1], have been reported by some individuals as a result of exposure to EMF emitted from mobile phones and their base stations. The World Health Organization (WHO) coined the term “idiopathic environmental intolerance attributed to electromagnetic fields” (IEI-EMF), which refers to symptoms reported by individuals after EMF exposure [2]. However, the association between the reported symptoms and EMF exposure cannot be explained scientifically [3]. Moreover, these symptoms can usually be attributable to alternative etiologies [4]. For instance, one study found that sleep disturbances reported by individuals with IEI-EMF were more likely to stem from other underlying psychological factors than EMF [5].

In recent years, the prevalence rates of IEI-EMF have varied worldwide [6–12]. A survey in California reported a prevalence rate of 3.2% [12], and another in Switzerland reported a prevalence rate of 5%. A review of the literature revealed that Taiwan had the highest prevalence rate of 13.3% (in 2007) [7], but it declined to 4.6% in 2012 [13]. Regardless of the wide range of prevalence rates reported worldwide, the WHO declared IEI-EMF to be a crucial global health concern in 2005 [3]. Patients can experience symptoms severe enough to hinder their daily activities and cause difficulty in maintaining social and familial relationships [14].

Studies have explored various EMF sources and exposure durations in an attempt to establish EMF exposure as the cause of IEI-EMF symptoms [15–23]. However, no association between EMF exposure and IEI-EMF has been confirmed. Nocebo effects have been reported to be associated with the idiopathic symptoms attributed to environmental risk factors [24]. Moreover, such effects have been reported in open provocation studies [25, 26], in which both individuals with IEI-EMF and controls exhibited significant scores on measurement scales for symptoms (such as anxiety, arousal, discomfort, relaxation, and fatigue) only when participants were informed that they were being exposed to EMF. Most double-blind provocation studies, in which nocebo effects could be minimized, did not report adverse health effects related to EMF exposure [23]. The failure of these studies to observe the effects, if any, associated with EMF exposure may be attributed to several reasons. For example, the intensity of EMF administered might be too weak to induce the effects, the effects might be too small for most participants to perceive, and the statistical power of the study might be too small to detect the effects [15, 27]. Consequently, whether humans, especially those with IEI-EMF, can accurately and consistently detect the presence of EMF remains controversial.

We conducted a provocation study in which participants were exposed to EMF at a power density of 1 W/m^2^, a high level that rarely (but still possibly) occurs in the real life. To minimize the confounding effects of the characteristics of the participants, we used a crossover experimental design in which the participants were blinded to the exposure status. In addition to assessing subjective symptoms, we monitored physiological changes that can be measured objectively. This study aimed to exam whether EMF exposure induces symptoms or physiological changes in individuals with IEI-EMF and even in those without IEI-EMF, to investigate whether people experience physiological changes while perceiving EMF exposure, and to identify individuals who could accurately discriminate between sham and real EMF exposure.

## Methods

### Participants

We recruited volunteers aged between 20 and 69 years through online advertisements and posters displayed in public places. Both individuals who self-reported IEI-EMF and who did not report IEI-EMF symptoms were recruited. We also included those with IEI-EMF who were referred by the Environmental Protection Administration of Taiwan. All participants were paid 1,500 New Taiwan Dollars (approximately 50 US Dollars) as compensation for their participation in the provocation test.

Because of safety concerns and to avoid interference from existing illness, we excluded participants with cancer, claustrophobia, pregnancy, coronary heart disease, or psychological disorders. We also excluded those who possessed catastrophic illness certification issued by the National Health Insurance or had pacemakers. We used the Electromagnetic Hypersensitive Questionnaire [28] to determine whether a participant should be placed in the IEI-EMF or control group. All participants with IEI-EMF were screened using the questionnaire to identify whether they were sensitive to either mobile phones or base stations.

### Administration of exposure

We applied a spherical near-field measurement system antenna (Model 700S-90, Nearfield Systems Inc., Torrance, CA, USA) and a 2-port microwave network analyzer (Agilent N5230C PNA-L, Keysight Technology Inc., Santa Rosa, CA, USA) to generate signals mimicking EMF from second-generation wireless telephony technology (2G) base stations of 900 MHz Global System for Mobile Communication (GSM) and 1800 MHz GSM as well as from third-generation wireless telephony technology (3G) base stations of 800 MHz GSM and 2100 MHz GSM. The peak power of each band was set at 0.25 W/m^2^ for an average combined power of 1 W/m^2^, which is one-tenth of the maximum environmental exposure recommended by the International Commission on Non-Ionizing Radiation Protection (ICNIRP) [29]. The provoked power density was determined using a survey meter (HI-2200 RF, ESCO Technologies Inc., St. Louis, MO, USA). The transmission antenna was covered with black fabric and hidden 50 cm behind the participants to avoid the effects of other confounders, such as acoustic cues or heat development. A fake antenna was set up in front of the participants to mimic the real base station.

**Fig. 1:**
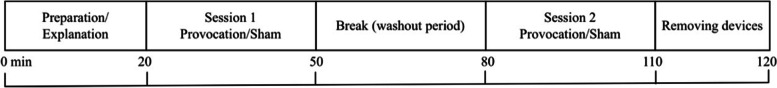
Provocation experiment procedure. Provocation and sham exposures were randomly assigned to either session 1 or session 2. The two sessions were separated by a 30-min break (washout period). The participants remained seated in a chair for the whole session, during which their physiological parameters were continuously recorded

We conducted the double-blind experiment in an anechoic laboratory (10.0 m × 6.5 m × 5.4 m) that was originally designed for antenna measurement and can thus block EMF from outside sources. Before entering the laboratory, each participant signed an informed consent form and completed the General Health Questionnaire (GHQ-28) [30] during the preparation/explanation period (Fig. 1). During this period, physiological parameters were recorded as baseline data, and the whole experiment process was re-explained to the participants. The participants were asked to adjust the seat to a position comfortable to them. After ensuring that the participants fulfilled the inclusion criteria and excluding candidates who were suffering from symptoms that we intended to study, we equipped them with physiological monitoring devices (MD-800, Comdek Technology Corp, Taiwan) to measure and record blood pressure (BP) and with the Holter monitor (DR200/HE, NorthEast Monitoring, Inc., Maynard, MA, USA) to record heart rate (HR) and HR variability (HRV).

The investigator drew lots placed in sealed envelopes to randomly assign the order of provocation (real) and sham sessions, and the investigator then handed the sealed envelope to the machine operator. Accordingly, the sham session was implemented after the provocation session in 49.8% of the experiments. After delivering the sealed envelope to the operator, the investigator ensured that the monitors functioned as intended before the experiment and then monitored the participant from outside the laboratory on a screen. The investigator entered the laboratory at 15-min intervals to ensure that the participant was filling out the questionnaire properly and that the monitor was functioning normally. The investigator and the participant were blinded to the sequence of provocation and sham sessions throughout the experiment. The duration of the provocation session, sham session, and washout period was 30 min each. During the provocation and sham sessions, the participants were asked to complete the Electromagnetic Hypersensitivity Questionnaire [28] and to record their perception of exposure and any symptoms they experienced, first after 15 min into the session and then again at the end of the session. All questionnaire items were based on a 5-point Likert scale. During the washout period, no EMF exposure was administered. The participants were required to stay awake and sit calmly in the chair throughout the procedure, including the washout period. They were monitored on a screen from outside the laboratory during the whole procedure to ensure that they stayed awake.

If the participants reported one of the sessions as being a provocation session with EMF exposure simply based on their knowledge that there were a provocation session and a sham session, their chance of being correct would be 50%. Therefore, those who correctly reported the exposure status were asked to undergo another round of the two-session test, and a third round if they reported the correct answers again. Participants who reported the exposure status correctly in three consecutive trials would be invited to participate in further tests to determine the lowest level of EMF they can detect.

### Physiological parameter measurements

The physiological parameters measured in this study included BP, HR, and HRV. BP parameters included systolic blood pressure (SBP), diastolic blood pressure (DBP), and mean arterial blood pressure (MAP). HRV refers to the ratio of low-frequency (LF, reflecting the activity of the sympathetic nervous system, the parasympathetic nervous system, and the baroreceptor reflex) to high-frequency (HF, reflecting the activity of the parasympathetic nervous system) pulses. The LF/HF ratio is the main indicator of HRV and represents the overall balance between the sympathetic nervous system and the parasympathetic nervous system [31]. All parameters were recorded at 5-min time intervals throughout all experimental sessions.

### Statistical analysis

We evaluated the differences in categorical variables between individuals with IEI-EMF and controls by using the χ^2^ test and those in continuous variables by using the two-sample *t* test. The consistency between the participants’ perception of exposure and their true exposure status was assessed using Cohen’s kappa test [32, 33]. We used McNemar’s test to evaluate the differences in self-reported symptoms between provocation and sham sessions. We applied linear mixed-effects models to analyze the data of physiological parameters recorded at 5-min intervals by using the LME command of the nlme package of R [34]. To minimize the effects of extreme values obtained as a result of measurement errors, we removed outliers by using the generalized extreme studentized deviate method [35].

All data analyses were conducted using R Version 3.3.2, SAS Version 9.3 and SPSS Version 17.0. All statistical tests were performed at a two-tailed significance level of 0.05.

## Results

A total of 58 individuals with IEI-EMF and 92 controls participated in this study (Table 1). The IEI-EMF group had higher proportions of women (63.8% vs. 29.3%, *p* < 0.01) and participants older than 40 years (34.4% vs. 9.8%, *p* < 0.01) than did the control group. The IEI-EMF group also had a higher proportion of participants who perceived their health status as “poor” or “very poor” (32.7% vs. 7.6%, *p* < 0.01). The distribution of occupation differed between the two groups (*p* = 0.03), with fewer students in the IEI-EMF group (21.1% vs. 42.4%). None of the differences between the two groups in other variables reached statistical significance (Table 1).

**Table 1:** Comparison between participants with idiopathic environmental intolerance attributed to electromagnetic fields (IEI-EMF) and normal controls

Variable	IEI-EMF *n* = 58 (%)	Control *n* = 92 (%)	*P*
Sex			< 0.01
Male	21 (36.2)	65 (70.7)	
Female	37 (63.8)	27 (29.3)	
Age (years)			< 0.01
20–29	18 (31.0)	64 (69.6)	
30–39	20 (34.5)	19 (20.7)	
40–49	14 (24.1)	7 (7.6)	
50–59	5 (8.6)	1 (1.1)	
60–69	1 (1.7)	1 (1.1)	
Education			0.22
High school	6 (10.3)	15 (16.3)	
Partial college	12 (20.7)	10 (10.9)	
Bachelor	28 (48.3)	53 (57.6)	
Graduate	12 (20.7)	14 (15.2)	
Occupation			0.03
Student	12 (21.1)	39 (42.4)	
Business	12 (21.1)	10 (10.9)	
Law	2 (3.5)	0 (0.0)	
Public service	7 (12.3)	6 (6.5)	
Service	12 (21.1)	20 (21.7)	
Others	12 (21.1)	17 (18.5)	
Self-perceived health status			< 0.01
Very poor	1 (1.7)	0 (0.0)	
Poor	18 (31.0)	7 (7.6)	
Average	25 (43.1)	51 (55.4)	
Good	12 (20.7)	29 (31.5)	
Excellent	2 (3.4)	5 (5.4)	
Baseline blood pressure (mmHg)			
Systolic (provocation)	121.1 ± 2.7	123.5 ± 1.7	0.44
Systolic (sham)	125.4 ± 2.9	124.2 ± 1.5	0.71
Diastolic (provocation)	76.9 ± 1.9	81.0 ± 1.3	0.07
Diastolic (sham)	80.4 ± 3.0	81.1 ± 1.4	0.84
Mean arterial (provocation)	92.0 ± 2.0	93.7 ± 1.4	0.50
M arterial (sham)	96.8 ± 2.9	94.9 ± 1.3	0.53
Baseline heart rate (/min)			
Provocation	77.7 ± 2.4	76.2 ± 1.2	0.57
Sham	78.5 ± 1.8	75.6 ± 1.1	0.17
Baseline heart rate variability			
LF (provocation)	0.70 ± 0.02	0.71 ± 0.02	0.63
LF (sham)	0.72 ± 0.02	0.71 ± 0.02	0.87
HF (provocation)	0.31 ± 0.02	0.29 ± 0.02	0.58
HF (sham)	0.29 ± 0.02	0.29 ± 0.02	0.90
LF/HF (provocation)	3.07 ± 0.32	3.24 ± 0.26	0.68
LF/HF (sham)	3.30 ± 0.32	3.40 ± 0.29	0.83

*LF* Low-frequency pulses, *HF* High-frequency pulses

The individuals with IEI-EMF were more likely than the controls to report perceiving the exposure regardless of whether the exposure actually existed (45.7% vs. 4.3%) or not (43.1% vs. 1.6%). Nonetheless, the accuracy of reporting the exposure was low in both groups, with a kappa value of 0.03 (95% confidence interval [CI]: -0.10–0.16) in the IEI-EMF group and a kappa value of 0.03 (95%CI: -0.01–0.06) in the control group. The results indicate that the participants in both groups were unable to accurately perceive their EMF exposure status. Most participants with IEI-EMF reported one session of the two sessions as either provocation or sham, and the distribution of answers was close to random. The participants in the control group reported perceiving the exposure in only approximately 3% of the occasions; therefore, the distribution of accurate answers considerably differed from the distribution of answers based on guesswork. In the IEI-EMF group, four participants reported the exposure status correctly in the first round of tests, but none of them could report the exposure status correctly again in the second round of tests. In the control group, one participant reported the exposure status correctly in the first round of tests but could not do so again in the second round of tests.

In the IEI-EMF group, 44 (75.9%) participants reported having symptoms during the provocation session, and 47 (81.0%) reported having symptoms during the sham session. The most commonly reported symptom during the provocation session was headache (52.6%), followed by fatigue (44.3%) and distraction (41.7%). The most commonly reported symptom during the sham session was headache (46.6%), followed by distraction (41.7%) and anxiety (41.3%). In the control group, 25 (27.2%) participants reported having symptoms during the provocation session, and 18 (19.6%) reported having symptoms during the sham session. The most commonly reported symptom during the provocation session was headache (6.5%), followed by distraction (4.9%) and fatigue (4.3%). The most commonly reported symptom during the sham session was headache (6.5%), followed by auditory symptoms such as tinnitus (5.4%) and fatigue (3.8%). Overall, the participants with IEI-EMF were more likely to report symptoms than the controls during both the provocation and sham sessions. In the IEI-EMF group, none of the reported symptoms were specifically related to EMF exposure (i.e., none of the symptoms were more frequently reported in the provocation sessions than in the sham sessions; Table 2). In the control group, one participant reported nausea in the provocation session but not in the sham session, and two such cases of desire to cough were observed. Headache, fatigue, distraction, anxiety, and auditory symptoms were the five most commonly reported symptoms in both the provocation and sham sessions. Differences in the symptoms reported by the participants in the IEI-EMF or control group between 15 min after the perception of exposure and the end of the session did not reach statistical significance, and a similar result was obtained for the differences in physiological parameters (data not shown).

**Table 2:** Symptoms reported by the participants

Symptom	IEI-EMF					Control				
	Sham	Provocation	P^+^/S^−^	S^+^/P^−^	OR (95% CI)	Sham	Provocation	P^+^/S^−^	S^+^/P^−^	OR (95% CI)
Nausea	18	21	6	3	2.0 (0.4–12.4)	0	1	1	0	NA
Distraction	24	24	5	5	1.0 (0.2–4.4)	3	3	1	1	1.0 (0.0–78.5)
Itch	20	14	1	7	0.1 (0.0–1.1)	3	2	2	3	0.7 (0.1–5.8)
Desire to cough	13	13	1	1	1.0 (0.0–78.5)	0	2	2	0	NA
Tingle	15	13	0	2	0.0 (0.0–5.3)	1	1	1	1	1.0 (0.0–78.2)
Anxiety	27	23	3	7	0.4 (0.1–0.9)	3	3	2	1	2.0 (0.1–118.0)
Headache	29	32	8	5	1.6 (0.5–6.2)	8	6	5	4	1.3 (0.3–6.3)
Diplopia	13	12	1	2	0.5 (0.0–9.6)	0	0	0	0	NA
Auditory symptoms	21	23	6	4	1.5 (0.4–7.2)	6	3	1	4	0.3 (0.0–2.5)
Eye itch	15	16	3	3	1.0 (0.2–18.0)	1	4	4	1	4.0 (0.4–197.0)
Palpitation	19	17	2	4	0.5 (0.1–3.5)	0	0	0	0	NA
Fatigue	23	27	8	4	2.0 (0.5–9.1)	4	5	3	2	1.5 (0.2–18.0)
Cold sweat	10	10	0	0	NA	0	0	0	0	NA

*P*^*+*^*/S*^*−*^ Symptom reported in the provocation session but not in the sham session, *S*^*+*^*/P*^*−*^ Symptom reported in the sham session but not in the provocation session, *OR* Odds Ratio, *CI* Confidence Interval, *NA* Not Available

By using the SBP, DBP, MAP, HR, and LF/HF ratio in the sham session as references, we found that the IEI-EMF group exhibited only slight changes in these physiological parameters during the provocation session, and none of the changes reached statistical significance (Table 3). Similarly, during the provocation session, the control group exhibited similar values of SBP, DBP, MAP, HR, and LF/HF ratio as those during the sham session. Because none of the participants were able to accurately report EMF exposure, we evaluated the effects of perceived exposure by comparing the physiological parameters during the session with perceived exposure with those during the session without perceived exposure of the same participant. The results revealed that except for the LF/HF ratio in the IEI-EMF group, the differences in the physiological parameters were generally larger between the sessions with and without perceived EMF exposure than between sessions with and without actual EMF exposure (Table 3). The participants in the IEI-EMF group exhibited similar SBP, DBP, MAP, HR, and LF/HF ratios when they reported perceiving EMF exposure and when they reported not perceiving the exposure. Although the control group also had similar SBP, DBP, MAP, and LF/HF ratios when they reported perceiving EMF exposure and when they reported not perceiving the exposure, their HR increased by 3.10 bpm (*p* = 0.03) when they reported perceiving the exposure compared with when they reported not perceiving the exposure.

**Table 3:** Changes in physiological parameters associated with provocation and perception of exposure to electromagnetic fields

Parameter	IEI-EMF			Control		
	Estimate	S.E	*P* value	Estimate	S.E	*P* value
Systolic blood pressure (mmHg)						
Provocation^a^	0.02	0.73	0.98	0.02	0.64	0.97
Perception^b^	-0.76	1.12	0.50	0.08	3.48	0.98
Diastolic blood pressure (mmHg)						
Provocation	0.40	0.69	0.56	0.35	0.62	0.57
Perception	-1.28	1.06	0.23	-5.58	3.31	0.09
Mean arterial pressure (mmHg)						
Provocation	0.05	0.70	0.94	-0.17	0.60	0.78
Perception	-0.26	1.06	0.80	-2.86	3.18	0.37
Heart rate (/min)						
Provocation	-0.65	0.84	0.44	0.40	0.30	0.18
Perception	-1.24	1.29	0.34	3.10	1.46	0.03^*^
LF/HF						
Provocation	0.19	0.11	0.10	0.09	0.09	0.35
Perception	-0.11	0.18	0.55	1.04	0.71	0.14

*SE* Standard Error, *LF* Low-frequency pulses, *HF* High-frequency pulses

^a^Linear mixed-effect model (LME) command of the nlme package of R, fixed factor: provocation, random-effect factor: individual, and time series

^b^Linear mixed-effect model (LME) command of the nlme package of R, fixed factor: perception (believing of EMF provoked), random-effect factor: individual, and time series

## Discussion

Our data suggested that short-term EMF exposure did not exert a significant effect on the BP of the participants in either the IEI-EMF or control group. This finding is in line with that of previous provocation studies, which have reported that short-term EMF exposure does not affect BP [17, 19, 36]. Our data also indicated that short-term EMF exposure does not affect HRV, which is also consistent with the findings of previous studies [37, 38], in which no change in HRV associated with EMF exposure was reported.

We searched Medline, PubMed, and Web of Science for research articles on double blind provocation studies that included participants with IEI-EMF by using the following keywords: “phone,” “base station,” “non-ionizing radiation,” “electromagnetic field,” “hypersensitivity,” “intolerance,” and “provocation.” A total of 12 articles that measured the physiological parameters of participants with IEI-EMF were published till the end of 2019 [17–20, 25, 26, 39–44]. In all the studies, participants could not accurately identify EMF exposure, and short-term exposure to EMF was not related to subjective symptoms or well-being. Specifically, three studies investigating BP [17, 19, 36] in a total of 150 participants with IEI-EMF and 112 controls did not observe an association between radio frequencies (900 MHz NMT, 900 MHz GSM, and 1800 MHz GSM) and SBP or DBP. Similarly, five studies investigating HR [17–19, 25, 26] in a total of 303 participants with IEI-EMF and 512 controls did not observe an association between EMF exposure and HR. These results are compatible with the findings of our study.

The 12 previous studies [17–20, 25, 26, 39–44] used exposure durations ranging from 10 min to 3 h, and the exposure intensity was lower than the intensity in the safety guidelines of the ICNIRP, which recommends a power density of 10 W/m^2^ for the general public [29] for radio frequencies ranging between 10 and 300 GHz. However, most of the studies were not conducted in EMF-shielding spaces; thus, participants were not shielded from environmental EMF. Therefore, one of the plausible reasons why the studies did not observe physiological changes induced by EMF is the interference caused by environmental EMF. In our study, the experiment was conducted in a laboratory that could block EMF from outside sources, thereby minimizing the interference of environmental EMF. Another plausible reason is that the dosage administered was too small to introduce an effect. Although the power of EMF we administered in the experiments was one-tenth of the ICNIRP maximum recommended environmental exposure value, it is much higher than those administered in the previous studies [17–20, 25, 26, 39–44], and such a high level is unlikely to be encountered under normal living conditions. In Taiwan, the average power density of 5,677 randomly selected spots for measurement by the Environmental Protection Administration between 1999 and 2021 was 0.23 W/m^2^, and only 18 (0.3%) of the measured values were higher than 1 W/m^2^ [46]. All the participants with IEI-EMF in our study reported experiencing symptoms in their daily lives, and the intensity of EMF exposure administered in our experiment was sufficient to provoke the symptoms if they are actually induced by EMF.

In our study, although four participants in the IEI-EMF group and one in the control group identified both the sham and provocation sessions correctly, none of them gave correct answers in the second trial. Accordingly, we determined that none of the participants could actually perceive the EMF exposure. This finding is consistent with the results of the previous studies [17–20, 25, 26, 39–44], all of which reported that participants could not accurately detect EMF exposure. This finding is also in line with the WHO’s statement in its Fact Sheet that there is no scientific basis for the association between IEI-EMF symptoms and EMF exposure [3]. We had planned to consider participants who reported the exposure status correctly in three consecutive two-session tests as being able to perceive the EMF exposure and to invite them to participate in further tests to determine the lowest level of EMF they can detect. Because none of the participants were able to even pass two consecutive tests, this part of the experiment protocol could not be executed.

Our study results also indicated that the symptoms reported by the participants were not related to EMF provocation. In fact, substantial overlaps were observed in the most commonly reported symptoms during the provocation and sham sessions between the IEI-EMF and control groups, and the odds ratios (ORs) of these symptoms were similar between the two groups. In the control group, the OR could not be calculated for five symptoms because no one reported these symptoms in the sham session without reporting them in the provocation session (Table 2). Of these symptoms, one participant reported nausea in the provocation session, but not in the sham session, and two such cases of desire to cough were observed. Because we used the Electromagnetic Hypersensitive Questionnaire to determine whether a participant should be placed in the IEI-EMF or control group, the symptoms reported by the participants in the control group were unlikely to be related to EMF exposure. Additionally, neither nausea nor desire to cough were related to EMF exposure in the IEI-EMF group. Furthermore, if there is an association between EMF and a given symptom, the OR in the IEI-EMF group should be larger than that in the control group. However, even when we combined data from the two groups under the assumption that they have the same OR, the *p* value of the OR associated with nausea was 0.34, and that of the OR associated with desire to cough was 0.63. Therefore, these symptoms could not be determined as associated with EMF exposure. These findings further support the inference that the symptoms reported by the participants were not related to EMF provocation. Some studies have proposed the use of models to illustrate how patients attribute idiopathic symptoms to various environmental factors. According to these models, in an attempt to identify the cause of their symptoms, patients are inclined to attribute these symptoms to environmental factors they have recently been exposed to. Their belief that these factors are causing their symptoms in turn leads them to develop further symptoms of intolerance towards the factors [4].

Previous provocation studies [25, 26] have found nocebo-like effects of EMF exposure on the physiological parameters of participants. To the best of our knowledge, however, this study is the first to explore the changes in the participants’ physiological parameters when they perceived exposure to EMF, irrespective of whether they have actually been exposed to EMF. The results revealed that except for the LF/HF ratio in the IEI-EMF group, the differences in physiological parameters were generally larger between the sessions with and without perceived EMF exposure than between the sessions with and without actual EMF exposure (Table 3). This indicates that the effects of perception on changes in physiological parameters are larger than those of actual EMF exposure. In addition, we found that the controls had elevated HR when they perceived EMF exposure, but this was not found in the participants with IEI-EMF. In fact, the participants with IEI-EMF had decreased (instead of elevated) HR when they perceived the exposure compared with when they did not, although the difference did not reach statistical significance. We speculate that the controls did not expect that they could perceive the existence of EMF and were thus surprised when they thought they did, which may have led to increases in HR. Further studies exploring the mechanism underlying this phenomenon might help elucidate the etiology of IEI-EMF.

This study has some limitations. First, according to the Working Group Meeting Report of the WHO Workshop on Electrical Hypersensitivity, IEI-EMF is defined as “symptoms that are experienced in proximity to, or during the use of, electrical equipment, and that result in varying degrees of discomfort or ill health in the individual and that an individual attributes to activation of electrical equipment” [2]. Therefore, IEI-EMF is defined on the basis of subjective symptoms and has no objective criteria for verification. However, this is a limitation of all IEI-EMF studies in general, not just of our study [45]. Second, it is possible that a certain level of EMF higher than that administered in this study may introduce remarkable physiological changes or symptoms in humans, but the purpose of this study was to assess physiological changes and symptoms associated with exposure to EMF in real life, not to test the limit of human beings. Moreover, exposing participants to such high doses of EMF might be considered unethical. Third, we did not include new technologies such as 4G and 5G because they were not widely used when this study was initiated, and further studies should be conducted to evaluate their potential effects. Fourth, we did not match age and sex in the selection of controls, and age and sex differences existed between the two groups. Although these factors might affect physiological parameters, the effects are generally unremarkable. For example, the diagnostic criteria for hypertension are generally the same, irrespective of age or sex. Nevertheless, additional studies with age and sex matching may address this issue directly. Fifth, we used OR to evaluate the associations between symptoms and EMF exposure. When no participant had a sham-positive/provocation-negative status, the OR could not be calculated; thus, the potential effects of EMF might not have been detected. However, this did not occur in our study; the only symptom with such data in the IEI-EMF group was “cold sweat,” and the number of participants in the IEI-EMF group with the provocation-positive/sham-negative status was also 0. Nevertheless, the number of participants in this study might not be sufficiently large to detect much rarer symptoms or smaller changes in physiological parameters. Furthermore, a washout period of 30 min might not be sufficiently long for some individuals with IEI-EMF, although we found no associations between the symptoms reported before the washout period and their existence during that the washout period.

In addition to conducting the provocation tests in a shielded laboratory and applying a high EMF exposure dose, this study had the advantage of a large sample size. Among the 12 previous studies assessed in the literature review, only one [39] included more participants with IEI-EMF (two more) than that in our study; moreover, our study included 32 more controls compared with this previous study. In addition, we collected information on both subjective symptoms and objective physiological parameters. Nevertheless, further studies are warranted to confirm our study findings, particularly the differences in physiological parameters between participants with IEI-EMF and controls when they perceived EMF exposure, which may provide insights into the etiology of IEI-EMF.

## Conclusions

The results of the provocation experiment indicate that short-term exposure to RF-EMF from mobile phone base stations would not affect the SBP, DBP, MAP, HR, or LF/HF ratio in individuals with or without IEI-EMF. We also found that none of the participants could accurately detect the EMF administered to them and that none of the symptoms reported by the participants with IEI-EMF were related to EMF exposure. Moreover, compared with the controls, the participants with IEI-EMF did not exhibit elevated HR levels when they perceived EMF exposure as the controls did. Further studies that explore changes in physiological parameters between individuals with and without IEI-EMF may provide insights into the etiology of IEI-EMF, and even that of IEI in general.

## Data Availability

The datasets used during the current study are available from the corresponding author on reasonable request.

## Abbreviations

BP: Blood pressure
DBP: Diastolic blood pressure
EHS: Electrohypersensitivity
EMF: Electromagnetic fields
GSM: Global System for Mobile Communication
HF: High frequency pulses
HR: Heart rate
HRV: Heart rate variability
IEI-EMF: Idiopathic environmental intolerance attributed to electromagnetic fields
LF: Low frequency pulses
MAP: Mean arterial blood pressure
SBP: Systolic blood pressure
